# Relationship between sleep disorders and the prognosis of neurological function after stroke

**DOI:** 10.3389/fneur.2022.1036980

**Published:** 2022-10-26

**Authors:** Yajing Zhang, Xiaoshuang Xia, Ting Zhang, Chao Zhang, Ran Liu, Yun Yang, Shuling Liu, Xin Li, Wei Yue

**Affiliations:** ^1^Department of Neurology, Tianjin Huanhu Hospital, Tianjin, China; ^2^Department of Neurology, The Second Hospital of Tianjin Medical University, Tianjin, China

**Keywords:** sleep disorders, neurological function, nocturnal total sleep time, sleepiness, OSA

## Abstract

**Objective:**

This study aims to investigate the effects of sleep disorders on the prognosis of neurological function after stroke and other factors affecting the prognosis after stroke.

**Method:**

We designed a cohort study. A total of 1,542 patients with their first stroke were hospitalized in the department of neurology of Tianjin Huanhu Hospital from 2015.6.1 to 2016.12.31. We recorded the personal histories of patients. The MMSE (mini-mental state examination), MoCA (Montreal Cognitive Assessment), HAMD (Hamilton Depression Scale), National Institutes of Health Stroke Scale (NIHSS) score, mRS (Modified Rankin Scale), BI (Barthel Index), PSQI (Pittsburgh Sleep Quality Index), ESS (Epworth Sleepiness Scale), Berlin questionnaire, and nocturnal TST (Total sleep time) were assessed before discharge, 3 months, 6 months, and 4 years (2019–2020) after stroke.

**Result:**

Low sleep quality (OR 2.019, 95%CI 1.199–3.398, *p* = 0.008), nocturnal TST (<7 h) (OR 4.060, 95%CI 1.494–11.034, *p* = 0.006), nocturnal TST (>8 h) (OR 5.928, 95% CI 2.134–16.464, *p* = 0.001) were risk factors for poor neurological function recovery at 3 months after stroke. Nocturnal TST (<7 h) (OR 13.042, 95%-CI 2.576–66.027, *p* = 0.002) and nocturnal TST (>8 h) (OR 11.559, 95%-CI 2.108–63.390, *p* = 0.005) were risk factors for poor neurological function at 6 months after stroke. Nocturnal TST (<7 h) (OR 2.668, 95% CI 1.250–5.698, *p* = 0.011) and nocturnal TST (>8 h) (OR 2.516, 95% CI 1.080–5.861, *p* = 0.033) were risk factors for poor neurological function at 4 years after stroke. High risk of OSA (HR 1.582, 95%CI 1.244–2.012, *p* < 0.001) was a risk factor for all-cause death in patients followed up for 4 years after stroke.

**Conclusion:**

Low sleep quality is associated with short-term poor neurological function after stroke. Unusual nocturnal TST (long or short) is associated with short-term or long-term poor neurological function after stroke. A high risk of OSA is associated with a higher risk of all-cause death after stroke.

## Introduction

Stroke is a common cerebrovascular disease. Approximately 16 million people worldwide experience their first stroke each year, of which ~5.7 million die, and another approximately five million people are left with a disability. Stroke is the third leading cause of death in men after heart disease and lung cancer, and the second leading cause of death in women (1).

Stroke is one of the leading causes of disability. Neurological function after stroke varies greatly in different individuals. The recurrence rate of stroke within 5 years is up to 17% (2). Thrombolytic drugs and endovascular therapy have revolutionized the status of acute stroke patients. However, for the vast majority of patients who remain disabled after treatment, there are significant challenges in improving neurological recovery and preventing stroke recurrence.

Stroke is the leading cause of death in China, accounting for 20% of all deaths every year. Understanding the risk factors for stroke is essential for better treatment. Hypertension, diabetes, atrial fibrillation, obesity, and smoking have been shown to be risk factors for stroke. The number of stroke patients, morbidity, mortality, and the associated social burden are high and increasing in China. The high burden of a stroke may be explained by the less significant changes in traditional risk factors and the persistent influence of the less recognized risk factors (3). However, there are few domestic studies on the relationship between sleep disorders and stroke. Screening for post-stroke sleep disorders has not yet become part of the standard of routine care, and screening coverage is low.

Sleep disorders after stroke are common, and about 20–78% of patients have sleep disorders (4). The role of sleep disorders in stroke outcomes has become a thorny problem. Sleep disorders can cause intracranial cerebral atherosclerosis or small vessel diseases (5–8).

Sleep disorders after stroke are underestimated and generally overlooked because of the lack of awareness of sleep disorders among stroke patients. Although polysomnography (PSG) is the gold standard for diagnosing or differentiating sleep disorders, PSG cannot be applied to all stroke patients due to its high cost and limited accessibility. Other tools are also needed to screen for sleep disorders, such as valid sleep questionnaires, such as the Pittsburgh Sleep Quality Index (PSQI), Epworth Sleepiness Scale (ESS), Sleepiness Scale, and Berlin questionnaire.

The purpose of this cohort study was to investigate the influence of longitudinal changes on sleep disorders (including sleep quality, sleepiness, nocturnal TST (Total sleep time), and obstructive sleep apnea (OSA), as measured by sleep questionnaires on neurological function and all-cause death in patients with stroke. Other factors affecting the neurological function of patients after stroke were also analyzed.

## Research object

A total of 1,542 patients with the first stroke, including cerebral infarction, TIA, and cerebral hemorrhage, were hospitalized in the department of neurology of Tianjin Huanhu Hospital from 2015.6.1 to 2016.12.31. (1) The inclusion criteria were as follows: ① the patients were all admitted 72 h after onset, and ② the diagnosis of ischemic stroke meets the diagnostic criteria of the 2014 Chinese Guidelines for diagnosis and treatment of acute ischemic stroke (9). The intracerebral hemorrhage diagnosis met the guidelines for the management of spontaneous intracerebral hemorrhage—a guideline for healthcare professionals from the American Heart Association/American Stroke Association (10). The diagnosis of TIA met the diagnostic criteria for definition and evaluation of transient ischemic attacks—a scientific statement for healthcare professionals from the American Heart Association/American Stroke Association Stroke Council; Council on Cardiovascular Surgery and Anesthesia; Council on Cardiovascular Radiology and Intervention; Council on Cardiovascular Nursing; and the Interdisciplinary Council on Peripheral Vascular Disease (11). ③ Patients who can provide written informed consent and are willing to follow the 4-year follow-up protocol. (2) The exclusion criteria were as follows: ① patients under 18 years of age; ② patients with obvious liver and kidney dysfunction, heart failure, severe infection, or malignant disease; ③ patients with a prior history of brain disease and cognitive impairment; ④ patients with specific genetic diseases; ⑤ patients with aphasia, apraxia, disturbance of consciousness, visual and hearing impairment, and other conditions that make it difficult to perform functional tests, as well as those who cannot accurately provide reliable information; and ⑥ patients with sleep disorders before stroke (we specifically excluded people with a previous diagnosis of OSA, heavy snoring, nocturnal TST <7 h and >8 h, and those who considered themselves to be extremely sleepy during the day).

## Research method

All enrolled patients underwent a detailed medical history, prior history, and personal history, as well as a detailed neurological examination, MRI, transcranial doppler, cervical ultrasound, MRA, or CTA. All patients underwent an MRI examination. According to MRI results, the lesions of the patients were divided into a dominant hemisphere and non-dominant hemisphere; large lesions (lesions larger than 4 cm in diameter or involving more than two lobes of the brain are called large lesions), brainstem lesions (midbrain, pons, and medulla oblongata lesions) and lesions in key areas (hippocampus, cingulate gyrus, and angular gyrus in cortical areas, thalamus, fornix, and basal ganglia in subcortical areas); multiple infarcts and non-multiple infarcts; microbleeds, and non-microbleeds. The medial temporal lobe atrophy rating scale (MTA) and the Fazekas scale were performed. The patients' arteries (internal carotid artery, middle cerebral artery, anterior cerebral artery, vertebral artery, and basilar artery) were divided into stenosis and non-stenosis based on vascular examination. Systolic blood pressure, diastolic blood pressure, and heart rate were recorded. Homocysteine (Hcy), fasting blood glucose (FBG), triglycerides (TG), cholesterol (TC), high-density lipoprotein (HDL), and low-density lipoprotein (LDL) were recorded. The educational level of the patients was recorded in detail. Patients with <6 years of education (illiterate and primary school) were in the low-level education group, and those with more than 6 years of education were in the high-level education group.

**Figure 1:**
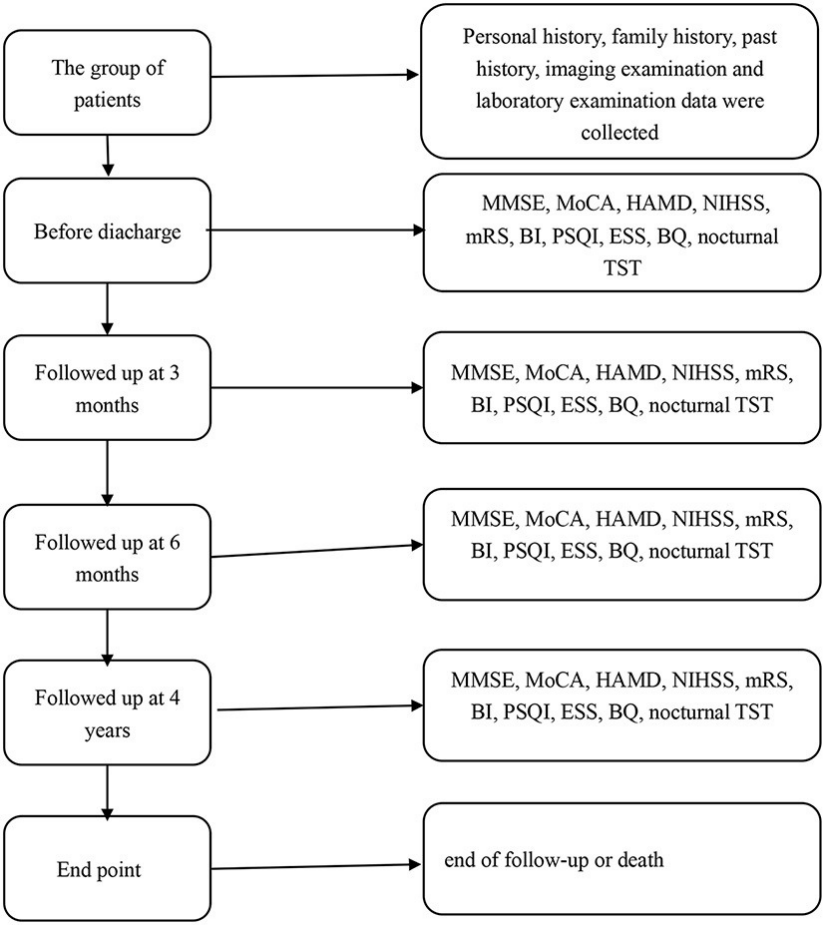
The flow chart of the research method.

Patients were given the detailed mini-mental state examination (MMSE) score, Montreal Cognitive Assessment (MoCA) score, Hamilton Depression Scale (HADM) score, National Institutes of Health Stroke Scale (NIHSS) score, Modified Rankin Scale (mRS) score, Barthel Index (BI), Pittsburgh Sleep Quality Index (PSQI) score, Epworth Sleepiness Scale (ESS) score, Berlin Questionnaire (BQ), and nocturnal total sleep time (TST) before discharge. The basic information of all patients was in Table 1. All patients were followed up at 3 months, 6 months, and 4 years (2019–2020) after stroke. During the follow-up, the above scores and questionnaires were conducted again to assess the relationship between the recovery of neurological function and the sleep status of the patients at that time. The endpoint was the end of follow-up, and the secondary endpoint was death. The flow chart of the research method was shown in Figure 1. The time of death and cause of death were recorded according to the patient's death certificate or medical record. Whether the patient had a wake-up stroke or early neurological deterioration (END) was recorded during the hospitalization. END was defined as an increase of ≥1 point in NIHSS motor score or ≥2 points in the total score during the 1st week after admission (12).

**Table 1:** Basic information of all patients.

Gender	Male	1,056 (68.5%)
	Female	486 (31.5%)
**Age (year)**		61.58 ± 10.71
Education	≤6 years	490 (31.8%)
	>6 years	1,052 (68.2%)
Stroke type	TIA	156 (10.1%)
	Cerebral infarction	1,275 (82.7%)
	Cerebral hemorrhage	111 (7.1%)
Hemisphere	Dominant hemisphere	763 (49.5%)
	Non-dominant hemisphere	779 (50.5%)
Lesion	Large lesion	77 (49.9%)
	Brain-stem lesion	291 (18.9%)
	Critical sites lesion	482 (31.3%)
	Other lesions	692 (44.9%)
Multiple	Yes	597 (38.7%)
	No	945 (61.3%)
Microbleeds	Yes	464 (30.1%)
	No	1,078 (69.9%)
MTA scores		2.59 ± 1.40
Fazekas scores		1.27 ± 0.952
ICA arteriostenosis	Yes	695 (45.1%)
	No	847 (54.9%)
MCA arteriostenosis	Yes	652 (42.3%)
	No	890 (57.7%)
ACA arteriostenosis	Yes	126 (8.2%)
	No	1,416 (91.8%)
VA arteriostenosis	Yes	415 (26.9%)
	No	1,127 (73.1%)
BA arteriostenosis	Yes	210 (13.6%)
	No	1,332 (86.4%)
Hypertension	Yes	1,128 (73.2%)
	No	414 (26.8%)
Coronary heart disease	Yes	334 (21.6%)
	No	1,208 (78.4%)
Diabetes	Yes	463 (30%)
	No	1,079 (70%)
Drinking	Yes	644 (41.8%)
	No	898 (58.2%)
Smoking	Yes	747 (48.4%)
	No	795 (51.6%)
Systolic pressure (mmHg)	151.74 ± 25.80	
Diastolic pressure (mmHg)	84.12 ± 13.88
Heart rate	68.68 ± 12.28
Hcy (umol/L)	17.79 ± 3.38
FBG (mmol/L)	6.47 ± 2.83
TG (mmol/L)	1.78 ± 1.15
TC (mmol/L)	6.02 ± 2.77
HDL (mmol/L)	1.02 ± 0.38
LDL (mmol/L)	7.17 ± 2.02
MoCA scores	18.04 ± 7.08
Depression	Yes	251 (16.3%)
	No	1,291 (83.7%)
Sleep quality	Low	942 (61.1%)
	High	600 (38.9%)
Sleepiness	Yes	383 (24.8%)
	No	1,159 (75.2%)
Nocturnal TST	7–8 h	379 (24.6%)
	<7 h	914 (59.3%)
	>8 h	249 (16.2%)
OSA	High risk	1,343 (87.1%)
	low risk	199 (12.9)
Wake-up stroke	Yes	593 (38.4%)
	No	949 (61.6%)
END	Yes	331 (21.5%)
	No	1,211 (78.5%)

**Figure 2:**
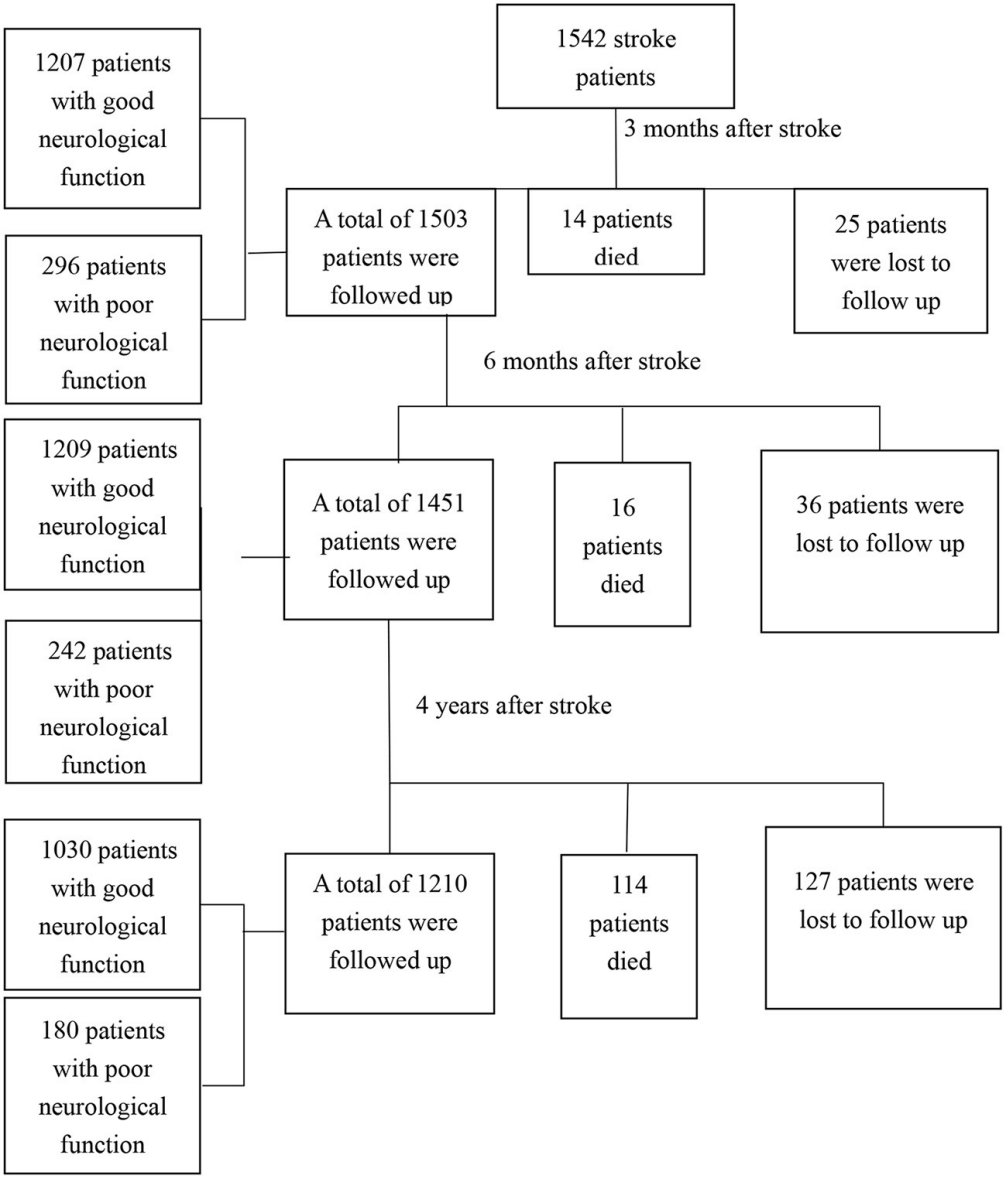
The flow chart of patients.

A total of 1,542 stroke patients were enrolled, and 188 patients were lost to follow-up. Among the 188 patients, 56 went to other places and could not cooperate with the follow-up, 103 withdrew from the follow-up, and 29 patients could not be contacted because their contact information had changed. A total of 1,354 patients, including 144 who died, completed follow-up. The flow chart of patients was shown in Figure 2.

All data were collected by neurologists and nurses through standard face-to-face questionnaires. Before the follow-up, all the investigators were given special training. The training included the purpose of the study, questionnaire management methods, questionnaire testing methods, and research procedures. We had specialists train our investigators on the cognitive scale, the neurologic scale, the depression scale, and the sleep disorders scale. The training was conducted in strict accordance with uniform guidelines, test procedures, and scoring standards. We needed to unify the instructions and operation procedures of each scale. After the training, the training personnel carried out practical operation drills and relevant assessments. Only qualified personnel could carry out the study. The personnel who assessed the scales at admission, 3 months after stroke, 6 months after stroke, and 4 years after stroke were different. In addition to bedside screening tests, we needed to ensure that tests were optimized and that patients could be evaluated in a neuropsychological testing room with an appropriate testing environment that eliminates test anxiety and allows adequate time and rest. Investigators were given adequate guidance and assistance during data collection.

This study has been ethically approved by the Tianjin Huanhu Hospital. The purpose of the study had been explained to all participants, confidentiality had been promised, and participants had been informed of their right to withdraw.

## Assessment of cognitive function

The Mini-mental State Scale (MMSE) and the Montreal Cognitive Assessment Scale (MoCA) are the most widely used cognitive function screening scales in a clinic. The MMSE, developed by Folstein in 1975, is one of the most widely used screening tools for cognitive impairment, and scores are easily influenced by education level. MMSE is poor at identifying early dementia, especially mild cognitive impairment. The MMSE consists of six aspects and a total of 30 questions. The Montreal Cognitive Assessment (MoCA) is a tool used to screen for cognitive impairment. The scale has been translated into many languages, and the popular versions in China are Beijing, Beijing-Guangzhou (Mandarin), Changsha, Cantonese, Hong Kong, and Taiwan. MoCA can detect the earliest stage of cognitive impairment. The MoCA measures eight cognitive domains: memory function, visuospatial function, executive function, attention, computation, language function, temporal orientation, and spatial orientation. The specificity of the Montreal Cognitive Scale (MoCA) for screening mild cognitive impairment (MCI) was 88.4%, and although it was slightly lower than the Mini-Mental State Examination (MMSE) (100%), its sensitivity to MCI was 92.4%, significantly better than that of the MMSE (24.2%). MoCA and MMSE can complement each other.

## Assessment of neurological function

The National Institutes of Health Stroke Scales (NIHSS) is a comprehensive acute stroke scale that major hospitals have widely adopted in China. The NIHSS score has 42 points and 11 items. It considers the symptoms of the anterior and posterior circulation and is an objective semi-quantitative stroke severity evaluation tool. It has been widely used in several international multicenter randomized controlled studies and is a recognized scoring system for the severity stratification of stroke patients with good reproducibility.

The Barthel Index (BI) was published by Florence Mahoney and Dorothea Barthel in the United States in 1965. The BI has comprehensive content, clear scoring, simple operation, high sensitivity, and good reliability and validity in face-to-face and telephone evaluations. In clinical applications, BI is used to assess baseline disability, assist in rehabilitation planning, and quantify functional changes after rehabilitation. BI is widely used in stroke clinical trials as a functional outcome measure. The Barthel Index includes ten items, which are divided into four functional grades (0, 5, 10, and 15 points), depending on the need for help and the degree of help, with a total score of 100 points. The higher the score, the more independent and less dependent it is. The mRS score is used to measure the neurological recovery status of patients after a stroke. It assesses the ability to live independently, including physical function, mobility, and daily life participation. It is divided into five levels. mRS ≤2 indicated a good prognosis of neurological function, and mRS>2 indicated a poor prognosis of neurological function. mRS at 3 and 6 months after stroke represented short-term neurological outcomes, and mRS at 4 years after stroke represented long-term neurological outcomes.

### Assessment of depression

Developed by Hamilton in 1960, the HAMD is the most commonly used self-measuring depression scale in the clinical evaluation of depression. The Hamilton Depression Scale (HAMD) has good reliability and validity. It can reflect the changes in depressive symptoms more sensitively and is considered one of the best evaluation tools in therapeutic research. The total score can better reflect the severity of depression; the milder the disease, the lower the total score. A total score of more than eight is considered depression.

### Assessment of sleep disorder

PSQI was developed in 1989 by Dr. Buysse, a psychiatrist at the University of Pittsburgh. It was used to evaluate sleep quality in clinical and basic research. Liu Xianchen translated the scale into Chinese in 1996 and conducted a study on its reliability and validity. The results showed that the scale also had high reliability and validity when applied to China. The PSQI is a self-reported questionnaire used to assess sleep quality. It contains 19 questions that indicate overall sleep quality. Each question is weighed into seven components on a 0–3 interval scale. PSQI <5 indicates high sleep quality, and PSQI ≥5 indicates low sleep quality.

BQ can be used to evaluate obstructive sleep apnea syndrome (OSAS) with a sensitivity of 60–70% and a specificity of 15–55% (13, 14).

BQ consists of ten questions divided into three categories: severity of snoring, excessive daytime sleepiness, and a history of hypertension or obesity. Patients with two or more positive categories were classified as “high-risk OSA patients,” and patients with one category of positive or asymptomatic groups were classified as “low-risk OSA patients (15).”

The ESS scale is a subjective evaluation of excessive daytime sleepiness (EDS) designed by the Epworth Sleep Research Center in Australia and is widely used in various sleep centers. It is one of the most practical sleep scales recognized internationally because of its accurate judgment and strong family self-test. The total score of ESS is 0–24, and an overall score of ≥10 indicates daytime sleepiness.

## Statistics

SPSS software (version 17.0) was used for data processing and analysis. Logistic univariate regression analysis was used to analyze the factors affecting the neurological function of patients at 3 months, 6 months, and 4 years after stroke. The dependent variable was neurological function, where mRS ≤2 was coded as 0, and mRS >2 was coded as 1. The significance of the univariate analysis and the factors associated with sleep disorders (sleep quality, sleepiness, nocturnal TST, and OSA) were included in the multivariate logistic regression analysis. The stepwise forward method was used to select the variables that were eventually included in the model. A survival analysis was performed for all patients who died during the 4-year follow-up. In the survival analysis, group data were presented as mean ± standard deviation for normal distributions and n (%) or median (interquartile range) for skewed distributions. Group comparisons were performed using the Student's ***t***-test, variance analysis of variance, or chi-square test as appropriate. Sleep disorders at admission were used as co-variables in the all-cause death-survival analysis of patients who died. Death was a dependent variable. Death was coded as one. Among the co-variables, stroke was classified into TIA, cerebral hemorrhage, and cerebral infarction, with cerebral hemorrhage as the reference category. Nocturnal TST was divided into 7–8 h, <7 h, and > 8 h, with 7–8 h as the reference category. The lesions were divided into the large brainstem, key sites, and other lesions; other lesions were used as references. A two-sided *P*-value <0.05 was considered statistically significant.

## Results

Three months after stroke, there were 1,207 patients with good neurological function (mRS ≤2), 296 patients with poor neurological function (mRS >2), and 14 patients (0.7%) with death. Univariate and multivariate logistic analyses of neurological function 3 months after stroke were shown in Table 2 (mRS ≤2 was coded as 0, mRS > two was coded as 1).

**Table 2:** Univariate and multivariate logistic analysis of neurological function three months after stroke.

		**Univariate analysis**		**Multivariate analysis**	
		**OR (95% CI)**	***p*-value**	**OR (95% CI)**	***p*-value**
Population information	Gender (male)	0.554 (0.420–0.731)	<0.001*	NA	
	Age	1.038 (1.025–1.052)	<0.001*	NA	
	low level of education	1.507 (1.141–1.992)	<0.001*	NA	
Stroke type	TIA	0.235 (0.114–0.483)	<0.001*	0.128 (0.031–0.539)	0.005
	cerebral infarction	0.552 (0.347–0.876)	0.012*	0.097 (0.040–0.237)	<0.001
Lesion characteristics	dominant hemisphere	1.392 (1.056–1.835)	0.021*	NA	
	Large lesion	4.361 (2.517–7.556)	<0.001*	3.992 (1.754–9.083)	0.001
	brainstem lesion	1.171 (0.781–1.754)	0.445	NA	
	critical sites lesion	1.610 (1.160–2.235)	0.004*	NA	
	multiple lesions	2.590 (1.949–3.442)	<0.001*	NA	
	MTA score	2.097 (1.801–2.442)	<0.001*	NA	
	Fazekas score	1.723 (1.551–1.913)	<0.001*	1.506 (1.266–1.790)	<0.001
	Microbleeds	3.077 (2.330–4.063)	<0.001*	NA	
arteriostenosis	ICA	1.713 (1.301–2.255)	<0.001*	NA	
	MCA	1.564 (1.189–2.057)	0.002*	NA	
	ACA	1.188 (0.738–1.914)	0.948	NA	
	VA	1.485 (1.106–1.993)	0.015*	NA	
	BA	2.122 (1.492–3.019)	0.021*	NA	
Risk factors	Hypertension	1.254 (0.911–1.725)	0.241	NA	
	coronary heart disease	0.726 (0.514–1.026)	0.068	NA	
	Diabetes	0.968 (0.718–1.304)	0.936	NA	
	Drinking	0.668 (0.503–0.889)	0.007*	NA	
	Smoking	0.691 (0.524–0.911)	0.010*	NA	
Blood pressure and heart rate	systolic pressure	1.013 (1.007–1.018)	<0.001*	NA	
	diastolic pressure	1.001 (0.991–1.011)	0.809	NA	
	heart rate	1.014 (1.003–1.026)	0.016*	1.019 (1.002–1.036)	0.028
laboratory examination	Hcy	0.996(0.984–1.009)	0.533	NA	
	FBG	0.982 (0.932–1.034)	0.405	NA	
	TG	0.825 (0.704–0.967)	0.017*	NA	
	TC	0.999 (0.991–1.006)	0.730	NA	
	HDL	1.124 (0.796–1.588)	0.502	NA	
	LDL	0.993 (0.963–1.023)	0.442	NA	
Patients status at three months after stroke	MoCA scores	0.883 (0.864–0.903)	<0.001*	NA	
	Depression	4.610 (3.287–6.464)	<0.001*	NA	
	low sleep quality	3.531 (2.594–4.806)	<0.001*	2.019 (1.199–3.398)	0.008
	Sleepiness	1.788 (1.338–2.390)	<0.001*	NA	
	nocturnal TST (<7h)	0.040 (0.001–1.754)	0.095	4.060 (1.494–11.034)	0.006
	nocturnal TST (>8h)	0.036 (0.000–2.745)	0.132	5.928 (2.134–16.464)	0.001
	High risk OSA	2.644 (1.551–4.506)	<0.001*	NA	
Characteristics of cases	Wake–up stroke	2.577 (1.910–3.478)	<0.001*	5.060 (3.300–7.758)	<0.001
	END	8.523 (6.160–11.793)	<0.001*	NA	

*p < 0.05, items with statistical significance in univariate analysis were included in the multivariate logistic regression analysis.

Univariate and multivariate logistic analyses of neurological function 3 months after stroke are shown in Table 2. Logistic univariate analysis showed gender, age, education, stroke type, dominant hemisphere, stroke lesion, multiple lesions, MTA score, Fazekas score, microbleeds, ICA stenosis, MCA stenosis, VA stenosis, BA stenosis, drinking, smoking, systolic blood pressure, heart rate, TG, MoCA scores, depression at 3 months, sleep quality, sleepiness, OSA, wake-up stroke, and END were statistically significant (*p* < 0.05). A multivariate logistic analysis of these factors was performed. In multivariate logistic analysis, large lesion (OR 3.992, 95% CI 1.754–9.083, *p* = 0.001), high Fazekas score (OR 1.506, 95% CI 1.266–1.790, *p* < 0.001), heart rate (OR 1.019, 95% CI 1.002–1.036, *p* = 0.028), low sleep quality (OR 2.019, 95% CI 1.199–3.398, *p* = 0.008), nocturnal TST (<7 h) (OR 4.060, 95% CI 1.494–11.034, *p* = 0.006), nocturnal TST (>8 h) (OR 5.928,95% CI 2.134–16.464, *p* = 0.001), wake-up stroke (OR 5.060, 95% CI 3.300–7.758, *p* < 0.001) were risk factors for poor neurological function recovery at 3 months after stroke. The neurological function recovery of TIA (OR 0.128, 95% CI 0.031–0.539, *p* = 0.005) and cerebral infarction (OR 0.097, 95% CI 0.040–0.237, *p* < 0.001) was better than that of intracerebral hemorrhage; thus, patients with intracerebral hemorrhage had poor neurological function.

6 months after the stroke, there were 1,209 patients with good neurological function, 242 patients with poor neurological function, and 16 patients who died. Univariate and multivariate logistic analyses of neurological function 6 months after stroke are shown in Table 3.

**Table 3:** Univariate and multivariate logistic analysis of neurological function at six months after stroke.

		**Univariate analysis**		**Multivariate analysis**	
		**OR (95% CI)**	***p*-value**	**OR (95% CI)**	***p*-value**
Population information	Gender (male)	0.542 (0.403–0.728)	<0.001*	NA	
	Age	2.718 (2.050–3.604)	<0.001*	NA	
	Low level of education	1.837 (1.367–2.468)	<0.001*	NA	
Stroke type	TIA	0.257 (0.115–0.573)	0.001*	0.107 (0.013–0.870)	0.037
	Cerebral infarction	0.654 (0.395–1.083)	0.099	0.104 (0.029–0.374)	0.001
Lesion characteristics	Dominant hemisphere	1.297 (0.965–1.743)	0.084	NA	
	Large lesion	3.300 (1.861–5.852)	<0.001*	NA	
	Brainstem lesion	1.113 (0.725–1.710)	0.624	NA	
	Critical sites lesion	1.471 (1.040–2.081)	0.029	NA	
	Multiple lesions	2.744 (2.025–3.717)	<0.001*	NA	
	MTA score	2.549 (2.150–3.022)	<0.001*	NA	
	Fazekas score	1.910 (1.700–2.145)	<0.001*	1.710 (1.320–2.215)	<0.001
	Microbleeds	3.479 (2.585–4.681)	<0.001*	NA	
Arteriostenosis	ICA	1.774 (1.319–2.386)	<0.001*	NA	
	MCA	1.624 (1.209–2.181)	0.002*	NA	
	ACA	1.375 (0.838–2.256)	0.300	NA	
	VA	1.558 (1.139–2.132)	0.008*	NA	
	BA	2.172 (1.498–3.149)	<0.001*	NA	
Risk factors	Hypertension	1.298 (0.917–1.838)	0.190	NA	
	Coronary heart disease	0.886 (0.620–1.265)	0.505	NA	
	Diabetes	1.021 (0.743–1.402)	0.899	NA	
	Drinking	0.652 (0.479–0.887)	0.008*	NA	
Blood pressure and heart rate	Smoking	0.693 (0.515–0.933)	0.017*	NA	
	Systolic pressure	1.015 (1.009–1.021)	<0.001*	NA	
	Diastolic pressure	0.998 (0.988–1.009	0.772	NA	
	Heart rate	1.014 (1.002–1.027)	0.023*	NA	
Laboratory examination	Hcy	0.999 (0.987–1.011)	0.844	NA	
	FBG	0.988 (0.935–1.044)	0.658	NA	
	TG	0.710 (0.583–0.865)	0.001*	NA	
	TC	0.999 (0.991–1.007)	0.768	NA	
	HDL	1.163 (0.817–1.656)	0.396	NA	
	LDL	0.992 (0.958–1.028)	0.486	NA	
Patients status at 6 months after stroke	MoCA score	0.862 (0.841–0.884)	<0.001*	NA	
	Depression	3.720 (2.639–5.243)	<0.001*	NA	
	low sleep quality	4.191 (3.036–5.786)	<0.001*	NA	
	Sleepiness	1.852 (1.192–2.877)	0.006*	NA	
	nocturnal TST (<7 h)	17.467 (8.499–35.897)	<0.001*	13.042 (2.576–66.027)	0.002
	nocturnal TST (>8 h)	15.913 (7.325–34.571)	<0.001*	11.559 (2.108–63.390)	0.005
	High risk OSA	3.459 (1.794–6.669)	<0.001*	NA	
Characteristics of cases	Wake–up stroke	2.913 (2.101–4.040)	<0.001*	NA	
	END	10.15 (7.156–14.422)	<0.001*	5.961 (3.213–11.061)	<0.001

*p < 0.05, items with statistical significance in univariate analysis were included in the multivariate logistic regression analysis.

Logistic univariate analysis showed gender, age, education, stroke type, stroke lesion, multiple lesions, MTA score, Fazekas score, microbleeds, ICA stenosis, MCA stenosis, VA stenosis, BA stenosis, drinking, smoking, systolic blood pressure, heart rate, TG, the score of MoCA at 6 months after stroke, depression at 6 months, sleep quality, sleepiness, OSA, nocturnal TST, wake-up stroke, and END were statistically significant (*p* < 0.05). A multivariate logistic analysis of these factors was performed.

Logistic multivariate analysis showed that patients with TIA (OR 0.107, 95% CI 0.013–0.870, *p* = 0.037) and cerebral infarction (OR 0.104, 95%-CI 0.029–0.374, *p* = 0.001) had better neurological function than patients with cerebral hemorrhage at 6 months after stroke; High Fazekas score (OR 1.710, 95% CI1. 320–2.215, *p* < 0.001), nocturnal TST (<7 h) (OR 13.042, 95%-CI 2.576–66.027, *p* = 0.002), nocturnal TST (>8 h) (OR 11.559, 95%–CI 2.108–63.390, *p* = 0.005) and END (OR 5.961 95% CI 3.213–11.061, *p* < 0.001) were risk factors for poor neurological function at 6 months after stroke.

4 years after the stroke, there were 1,030 patients with good neurological function, 180 patients with poor neurological function, and 114 patients who died. Univariate and multivariate logistics of neurological function 4 years after stroke are shown in Table 4.

**Table 4:** Univariate and multivariate logistic of neurological function at 4 years after stroke.

		**Univariate analysis**		**Multivariate analysis**	
		**OR (95% CI)**	***p*-value**	**OR (95% CI)**	***p*-value**
Population information	Gender (male)	0.590 (0.426–0.818)	0.002*	NA	
	Age	1.049 (1.032–1.066)	<0.001*	NA	
	Low level of education	1.812 (1.309–2.508)	<0.001*	NA	
Stroke type	TIA	0.424 (0.148–1.215)	0.110	NA	
	Cerebral infarction	1.503 (0.762–2.967)	0.240	NA	
Lesion characteristics	Dominant hemisphere	1.315 (0.952–1.817)	0.097	NA	
	Large lesion	2.7561.452–5.230)	0.002*	NA	
	Brainstem lesion	0.954 (0.595–1.528)	0.844	NA	
	Critical sites lesion	1.335 (0.919–1.938)	0.129	NA	
	Multiple lesions	2.793 (2.012–3.877)	<0.001*	NA	
	MTA score	2.842 (2.336–3.457)	<0.001*	1.649 (1.208–2.251)	0.002
	Fazekas score	2.076 (1.816–2.374)	<0.001*	1.323 (1.068–1.639)	0.010
	Microbleeds	5.349 (3.829–7.473)	<0.001*	NA	
Arteriostenosis	ICA	1.649 (1.191–2.282)	0.002*	1.858 (1.195–2.889)	0.006
	MCA	1.397 (1.009–1.934)	0.043*	NA	
	ACA	1.345 (0.761–2.376)	0.307	NA	
	VA	1.411 (0.991–2.008)	0.056	NA	
	BA	2.269 (1.491–3.455)	<0.001*	NA	
Risk factors	Hypertension	1.263 (0.864–1.846)	0.939	NA	
	Coronary heart disease	0.673 (0.435–1.041)	0.075	NA	
	Diabetes	1.006 (0.708–1.430)	0.604	NA	
	Drinking	0.721 (0.516–1.005)	0.556	NA	
Blood pressure and heart rate	Smoking	0.832 (0.602–1.151)	0.623	NA	
	Systolic pressure	1.014 (1.008–1.020)	<0.001*	NA	
	Diastolic pressure	0.999 (0.988–1.011)	0.899	NA	
	Heart rate	1.015 (1.001–1.029)	0.031*	NA	
Laboratory examination	Hcy	0.989 (0.970–1.008)	0.233	NA	
	FBG	0.995 (0.938–1.056)	0.868	NA	
	TG	0.888 (0.751–1.051)	0.168	NA	
	TC	0.984 (0.853–1.136)	0.552	NA	
	HDL	0.909 (0.559–1.480)	0.702	NA	
	LDL	0.872 (0.713–1.066)	0.492	NA	
Patients status at 4 years after stroke	MoCA score	0.821 (0.797–0.846)	<0.001*	NA	
	Depression	4.535 (3.260–6.308)	<0.001*	5.226 (3.353–8.147)	<0.001
	Low sleep quality	3.633 (2.572–5.133)	<0.001*	NA	
	Sleepiness	2.600 (1.865–3.626)	0.006*	NA	
	Nocturnal TST (<7 h)	10.133 (5.530–18.568)	<0.001*	2.668 (1.250–5.698)	0.011
	Nocturnal TST (>8 h)	10.412 (5.287–20.502)	<0.001*	2.516 (1.080–5.861)	0.033
	High risk OSA	2.355 (1.278–4.338)	0.006*	NA	
Characteristics of cases	Wake–up stroke	3.001 (2.166–4.158)	<0.001*	NA	
	END	10.628 (7.485–15.090)	<0.001*	5.226 (3.353–8.147)	<0.001

*p < 0.05, items with statistical significance in univariate analysis were included in the multivariate logistic regression analysis.

Logistic univariate analysis showed gender, age, education, stroke lesion, multiple lesions, MTA score, Fazekas score, microbleeds, ICA stenosis, MCA stenosis, BA stenosis, systolic blood pressure, heart rate, MoCA score, depression, sleep quality, sleepiness, OSA, nocturnal TST, wake-up stroke, and END were statistically significant (*p* < 0.05). A multivariate logistic analysis of these factors was performed.

Logistic multivariate analysis showed that MTA score (OR 1.649,95% CI 1.208–2.251, *p* = 0.002), Fazekas score (OR 1.323, 95% CI 1.068–1.639, *p* = 0.01), ICA arteriostenosis (OR 1.858,95% CI 1.195–2.889, *p* = 0.006), depression (OR 5.226, 95% CI 3.353–8.147, *p* < 0.001), nocturnal TST (<7 h) (OR 2.668, 95% CI 1.250–5.698, *p* = 0.011), nocturnal TST (>8 h) (OR 2.516,95% CI 1.080–5.861, *p* = 0.033), END (OR 5.226, 95% CI 3.353–8.147, *p* < 0.001) were risk factors for poor neurological function at 4 years after stroke.

At the end of the 4-year follow-up, of the 1,354 patients who completed the follow-up, 1 210 patients were still alive (89.4%), and 144 patients had died (10.6%). Basic information on dead patients and survivors 4 years after stroke is shown in Table 5. Univariate and multivariate COX analysis of the influencing factors of all-cause death is shown in Table 6 (survivors were coded 0, and dead patients were coded 1).

**Table 5:** Basic information of dead patients and survivors 4 years after stroke.

		**Survivors**	**Died patients**	***t*/χ^2^**	***p***
Gender	Male	837 (69.2%)	90 (62.5%)	2.654	0.103
	Female	373 (30.8%)	54 (37.5%)		
Age (year)		60.45 ± 10.33	70.62 ± 9.49	−11.394	<0.001
Education	≤6 years	370(30.6%)	58(40.3%)	5.600	0.018
	>6 years	840(69.4%)	86 (59.7%)		
Stroke type	TIA	121 (10.0%)	18 (12.5%)	0.919	0.632
	Cerebral infarction	1,000 (82.6%)	115 (79.9%)		
	Cerebral hemorrhage	89 (7.4%)	11 (7.6%)		
Hemisphere	Dominant hemisphere	567 (50.2%)	61 (44.2%)	1.756	0.185
	Non-dominant hemisphere	563 (49.8%)	77 (55.8%)		
Lesion	Large lesion	78 (6.4%)	13 (9.0%)	1.594	0.661
	Brain-stem lesion	234 (19.3%)	29 (20.1%)		
	Critical sites lesion	374 (30.9%)	41 (28.5%)		
	Other lesions	524 (43.3%)	61 (42.4%)		
Multiple	Yes	422 (37.9%)	68 (47.2%)	4.607	0.032
	No	690 (62.1%)	76 (52.8%)		
Microbleeds	Yes	331 (27.4%)	76 (52.8%)	39.560	<0.001
	No	879 (72.6%)	68 (47.2%)		
MTA scores		1.19 ± 0.92	1.96 ± 0.93	−9.642	<0.001
Fazekas scores		2.49 ± 1.38	3.42 ±1.32	−7.751	<0.001
ICA arteriostenosis	Yes	527 (43.6%)	82 (56.9%)	9.324	0.002
	No	683 (56.4%)	62 (43.1%)		
MCA arteriostenosis	Yes	504 (41.7%)	74 (52.3%)	4.986	0.026
	No	706 (58.3%)	70 (47.7%)		
ACA arteriostenosis	Yes	115 (9.5%)	18 (12.5%)	1.304	0.254
	No	1,095 (90.5%)	126 (87.5%)		
VA arteriostenosis	Yes	317 (26.2%)	60 (41.7%)	15.326	<0.001
	No	893 (73.8%)	84 (58.3%)		
BA arteriostenosis	Yes	167 (13.8%)	40 (27.8%)	19.409	<0.001
	No	1,043 (86.2%)	104 (72.2%)		
Hpertension	Yes	882 (72.9%)	102 (70.8%)	0.275	0.600
	No	328 (27.1%)	42 (29.2%)		
Coronary heart disease	Yes	243 (20.1%)	52 (36.1%)	19.401	<0.001
	No	967 (79.9%)	92 (63.9%)		
Diabetes	Yes	366 (30.2%)	46 (31.9%)	0.175	0.676
	No	844 (69.8%)	98 (68.1%)		
Drinking	Yes	536 (44.3%)	34 (23.6%)	22.592	<0.001
	No	674 (55.7%)	110 (76.4%)		
Smoking	Yes	612 (50.6%)	46 (31.9%)	17.887	<0.001
	No	598 (49.4%)	98 (68.1%)		
systolic pressure (mmHg)		150.97 ± 25.90	157.77 ± 24.55	−3.008	0.003
Diastolic pressure (mmHg)		84.53 ± 14.09	80.79 ± 11.77	3.534	0.001
Heart rate		68.70 ± 12.35	70.00 ± 11.77	−1.189	0.234
Hcy (umol/L)		18.31 ± 20.25	14.13 ± 9.34	1.579	0.115
FBG (mmol/L)		6.45 ± 2.82	6.72 ± 2.91	−1.054	0.292
TG (mmol/L)		1.804 ± 1.18	1.61 ± 0.88	1.788	0.074
TC (mmol/L)		6.02 ± 26.24	5.99 ± 6.20	0.013	0.990
HDL (mmol/L)		1.01 ± 0.39	1.04 ± 0.29	−0.825	0.409
LDL (mmol/L)		7.65 ± 90.33	3.45 ± 0.87	0.541	0.589
MoCA scores		18.50 ± 6.93	14.43 ± 7.28	6.695	<0.001
Depression	Yes	192 (15.9%)	30 (20.8%)	2.315	0.128
	No	1,018 (84.1%)	114 (79.2%)		
Sleep quality	Low	719 (59.4%)	108 (75.0%)	13.137	<0.001
	High	491 (40.6%)	36 (25.0%)		
Sleepiness	Yes	294 (24.3%)	42 (29.2%)	1.635	0.201
	No	916 (75.7%)	102 (70.8%)		
Nocturnal TST	7–8 h	322(26.6%)	12(8.3%)	23.508	<0.001
	<7 h	694(57.4%)	106(73.6%)		
	>8 h	194(16.0%)	26(18.1%)		
OSA	High risk	1,031 (85.1%)	143 (99.3%)	22.458	<0.001
	low risk	181 (14.9%)	1 (0.7%)		
Wake-up stroke	Yes	511 (42.2%)	57 (39.6%)	0.371	0.543
	No	699 (57.8%)	87 (60.4%)		
END	Yes	263 (21.7%)	29 (20.1%)	0.194	0.660
	No	947 (78.3%)	115 (79.9%)		

**Table 6:** Univariate and multivariate COX analysis of the influencing factors of all-cause death (survivors were coded 0, and dead patients were coded 1).

		**Univariate COX analysis**	**Multivariate COX analysis**		
		**OR (95% CI)**	***p*-value**	**OR (95% CI)**	***p*-value**
Population information	Gender (male)	0.754 (0.541–1.052)	0.096	NA	
	Age	1.098 (1.080–1.117)	<0.001*	NA	
	low level of education	1.505 (1.084–2.089)	0.015*	NA	
Stroke type	TIA	1.180 (0.577–2.413)	0.651	NA	
	Cerebral infarction	0.825 (0.455–1.494)	0.525	NA	
Lesion characteristics	Dominant hemisphere	0.803 (0.574–1.123)	0.199	NA	
	Large lesion	1.531 (0.783–2.996)	0.213	NA	
	Brainstem lesion	1.057 (0.665–1.678)	0.815	NA	
	Critical sites lesion	0.938 (0.623–1.412)	0.758	NA	
	Multiple lesions	1.435 (1.020–2.020)	0.038*	NA	
	MTA score	2.230 (1.879–2.647)	<0.001*	NA	
	Fazekas score	1.550 (1.381–1.740)	<0.001*	NA	
	Microbleeds	2.795 (2.024–3.859)	<0.001*	NA	
Arteriostenosis	ICA	1.967 (1.407–2.750)	<0.001*	1.871 (1.192–2.937)	0.007
	MCA	1.488 (1.074–2.064)	0.017*	NA	
	ACA	1.682 (1.026–2.756)	0.039*	NA	
	VA	2.018 (1.449–2.811)	<0.001*	NA	
	BA	2.609 (1.811–3.757)	<0.001*	1.725 (1.095–2.717)	0.019
Risk factors	Hypertension	0.847 (0.595–1.205)	0.356	NA	
	Coronary heart disease	2.126 (1.521–2.971)	<0.001*	NA	
	Diabetes	1.125 (0.797–1.587)	0.503	NA	
	Drinking	0.422 (0.290–0.614)	<0.001*	NA	
Blood pressure and heart rate	Smoking	0.484 (0.343–0.684)	<0.001*	NA	
	Systolic pressure	1.007 (1.003–1.012)	0.002*	NA	
	Diastolic pressure	0.982 (0.970–0.994)	0.002*	NA	
	Heart rate	1.008 (0.995–1.022)	0.248	NA	
Laboratory examination	Hcy	0.980 (0.955–1.006)	0.125	NA	
	FBG	1.028 (0.973–1.086)	0.324	NA	
	TG	0.836 (0.691–1.012)	0.066	NA	
	TC	1.000 (0.993–1.007)	0.991	NA	
	HDL	1.146 (0.815–1.613)	0.434	NA	
	LDL	0.997 (0.982–1.013)	0.741	NA	
Patients status at admission	MoCA score	0.931 (0.911–0.951)	<0.001*	NA	
	Depression	1.462 (0.988–2.162)	0.057	NA	
	NIHSS score	1.117 (1.069–1.167)	<0.001*	NA	
	Low sleep quality	1.899 (1.313–2.746)	0.001*	NA	
	Sleepiness	1.304 (0.917–1.856)	0.139	NA	
	High risk OSA	25.185 (3.223–196.810)	0.002*	1.582 (1.244–2.012)	<0.001
	Nocturnal TST (<7 h)	3.892 (2.142–7.070)	<0.001*	NA	
	nocturnal TST (>8 h)	3.755 (1.910–7.385)	<0.001*	NA	
Characteristics of cases	Wake–up stroke	1.028 (0.986–1.072)	0.188	NA	
	END	0.916 (0.195–4.315)	0.912	NA	

*p < 0.05, items with statistical significance in univariate analysis were included in the multivariate logistic regression analysis.

Univariate and multivariate COX analysis of the influencing factors of all-cause death (survivors were coded 0 and dead patients were coded 1) is shown in Table 6. Univariate COX analysis revealed age, education, multiple lesions, MTA score, Fazekas score, microbleeds, ICA stenosis, MCA stenosis, ACA stenosis, VA stenosis, BA stenosis, coronary heart disease, drinking, smoking, systolic blood pressure, diastolic blood pressure, MoCA score, NIHSS score, sleep quality, OSA, and nocturnal TST were statistically significant (*p* < 0.05). Multivariate COX analysis of these factors showed that ICA stenosis (HR 1.871, 95% CI 1.192–2.937, *p* = 0.007), BA stenosis (HR 1.725, 95% CI 1.095–2.717, *p* = 0.019), high risk of OSA (HR 1.582, 95% CI 1.244–2.012, *p* < 0.001) were risk factors for all-cause death in patients followed up for 4 years after stroke.

## Discussion

Our study found that low sleep quality was a risk factor for poor neurological function 3 months after stroke. However, there was no significant effect on long-term neurological recovery after stroke. This study used a PSQI questionnaire to assess sleep quality. In clinical work, the PSQI questionnaire is a simple and effective method to assess sleep quality. Insomnia is associated with an increased incidence of stroke and a poor prognosis. A Taiwan study of 21,438 patients with insomnia and 64,314 age - and gender-matched patients without insomnia observed a 54% increased risk of stroke in patients with insomnia over a 4-year follow-up (16). There are limited data on the relationship between neurological function recovery after stroke and the effect of sleep quality, especially from large prospective cohort studies. One study found that a lower sleep score (poor sleep profile) using PSQI is associated with a higher risk of coronary heart disease (17). One study showed that post-stroke sleep disturbance is associated with poorer outcomes from strokes (18).

Our study found that nocturnal TST (<7 h) and nocturnal TST (>8 h) were risk factors for short-term or long-term poor neurological function after stroke. A study of 123 inpatient rehabilitations found that, after adjusting for confounding factors, insomnia was associated with reduced ability to do daily living and poor recovery (19). Sleep disorders can negatively affect health in several ways, but not directly. Laboratory studies have shown that short sleep duration disrupts glucose metabolism, which increases the risk of diabetes (20). Short sleep increases blood pressure, C-reactive protein, cortisol levels, and sympathetic nervous system activity, which can lead to hypertension and cerebrovascular disease. Studies have observed a J-shaped association between sleep duration and stroke, and people with a sleep duration of 7 h have the lowest risk of stroke (21). People who sleep longer have a higher risk of stroke than people who sleep shorter, and the risk of stroke increases by 13% for each 1-h increase in sleep time over 7 h (21). One study found that nocturnal TST of 7–8 h can reduce the risk of stroke, and nocturnal TST ≥9 h is associated with an increased risk of stroke in 45–65-year-old people (22). Studies have shown a “U” type relationship between TST and stroke (23). Long nocturnal TST is a potential risk factor for stroke, which may be related to sympathetic activation. Long nocturnal TST increases the activity of orexin and inflammatory response, leading to elevated lipid levels and an increased risk of stroke. The increase in sleep time may be due to an initial lack of sleep, followed by more sleep to compensate for the previous lack of sleep. However, “lie-in” cannot alleviate the metabolic disorders caused by insufficient sleep and may even aggravate the metabolic disorders (24). “Sleep deprivation” can have adverse effects, but “sleep compensation” does not ameliorate those effects. Prolonged sleep duration may be a sign of subclinical disease.

The EES scale is widely used in the field of sleep medicine as a subjective measure of sleepiness in patients. Sleepiness is the inability to remain awake and alert during daytime waking periods, leading to the inadvertence of drowsiness or sleep. It is estimated that ~20% of adults experience sleepiness. Daytime sleepiness is a major public health problem because it has been linked to cognitive impairment, traffic accidents, medical negligence, and reduced productivity. In China, daytime napping is often considered a healthy lifestyle, especially for the elderly. However, studies have shown a potential relationship between daytime napping and stroke incidence (25). Daytime sleepiness, characterized by daytime naps, is considered to be an indicator of poor sleep quality or health disorders. In two studies with 9,095 participants followed up for over 5.1 years (208 stroke patients), sleepiness (ESS score ≥10) was found to be a predictor of stroke after adjusting for confounders such as age, sex, vascular risk factors, and comorbidities (25, 26). In a study of 213 stroke patients treated in a rehabilitation center, sleepiness was found to affect post-stroke rehabilitation. Patients with sleepiness had a higher rate of disability and a higher rate of transferring to a nursing home at discharge (27). The effects of sleepiness on stroke may include several aspects. First, sleepiness may be a manifestation of sleep disorders such as insufficient sleep duration or circadian rhythm disturbances, which can increase the risk of stroke. Second, sleepiness may be caused by an underlying disease that may be a risk factor for stroke. In addition, sleepiness may lead to an increase in caffeine intake, which disrupts nocturnal sleep and increases daytime sleepiness. Sports activities can improve sleep quality and reduce sleepiness, but sleepiness can lead to insufficient energy to participate in regular sports activities, which can increase the risk of stroke. However, many previous studies have shown that sleepiness is associated with poor outcomes after stroke. In our study, the effect of sleepiness assessed by the ESS scale on neurological function after stroke was not found. Further confirmation with large-scale objective measures is needed.

Studies have shown that OSA precedes stroke rather than being the consequence of stroke. A prospective study on OSA found that the incidence of OSA was consistent before and after stroke, suggesting that OSA is a common inducing factor (28). Previous studies have found no association between the prevalence or severity of OSA and stroke subtypes or stroke severity. The Wisconsin cohort study, which included 1,189 healthy participants, showed that sleep-related apnea-hypopnea index (AHI) ≥20/h was associated with an increased risk of stroke over the next 4 years (29). In a community-based study of 5,422 healthy participants without stroke who were assessed for PSG with a mean follow-up of 8.7 years, participants with an AHI>15/h were shown to have a 30% higher risk of stroke (30). A meta-analysis of 17 population-based prospective cohort studies showed that moderate to severe OSA significantly increased the risk of stroke (RR 2.02, 95% CI 1.4–2.9) (31). OSA is not only an independent risk factor for stroke but is also associated with a poor prognosis of stroke and an increased risk of stroke recurrence and death. OSA can induce stroke recurrence, increase the risk of death, and have a negative impact on the prognosis of stroke. In a prospective cohort study of 166 patients with ischemic stroke, 96 (58%) patients with AHI ≥20/h were provided with continuous positive airway pressure (CPAP) (32). 7 years later, patients with more severe OSA who could not tolerate CPAP had a significantly increased risk of non-fatal cerebrovascular disease (CVD) and recurrent stroke compared with patients without OSA or with mild OSA supported by CPAP. OSA may be a predictor of poor neurological function after stroke. OSA increases the risk of short-term neurological decline and long-term neurological dependence. In a study of 41 patients with acute ischemic stroke, OSA severity was associated with acute stroke severity and mRS score at discharge (33). The mechanism by which OSA causes poor neurological function after stroke is unclear, but it may be related to the damage of OSA to brain tissue, including the effect of sleep fragmentation on cognitive function and the effect of intermittent hypoxemia on ischemia and neuroplasticity. There are little data on the long-term effects of OSA treatment in patients with ischemic stroke or TIA. However, some current studies have shown that early OSA treatment can improve the prognosis of stroke, including stroke severity, neurological status, and recurrence (34). OSA may affect the prognosis of stroke in several ways. The direct effects of apnea are decreased oxygen saturation, sympathetic activation, and elevated blood pressure. In addition, OSA is associated with insulin resistance, dyslipidemia, elevated systemic inflammation, hypercoagulability, and impaired endothelial function, which may influence the prognosis of stroke. A prospective 10-year follow-up study of stroke rehabilitation patients found that OSA patients had a significantly higher risk of death than controls (35). Another study found that OSA patients had a 2-fold increased risk of stroke or all-cause death over 3.4 years, independent of known vascular risk factors, and the risk of stroke or all-cause death increased with the increase of OSA severity (36). Our study did not find an association between the high risk of OSA and poor neurological function after stroke but found that the high risk of OSA was a risk factor for all-cause death after stroke.

Early neurological function is also poor when there is intracerebral hemorrhage, large lesions, or multiple lesions. These patients have more severe neurological impairment at the onset and a poor prognosis of early neurological function. Patients with high MTA scores and Fazekas scores had a poor neurological outcome due to a higher percentage of patients with high MTA scores and Fazekas scores with cognitive impairment, which is closely associated with poor neurological outcomes. Stroke patients with ICA stenosis, BA stenosis, and END have a severe illness, poor prognosis, and high mortality, so ICA stenosis, BA stenosis, and END are associated with poor neurological outcomes.

## Conclusion

Low sleep quality, nocturnal TST (<7 h), and nocturnal TST (>8 h) were associated with poor neurological function after stroke. A high risk of OSA was associated with a higher risk of all-cause death after stroke.

### Limitations

We did not diagnose sleep disorders through objective tests but through the PSQI questionnaire, ESS questionnaire, Berlin questionnaire, and STOPBANG questionnaire, which may have potential deviations. However, many studies have proven these scales to be reliable screening measures for sleep disorders. More importantly, our investigators are highly trained, and the study also included comprehensive information on sleep disorders and longitudinal follow-up, which may make the results more accurate.

In future research, we need to further improve the evaluation methods for sleep disorders and use objective evaluation methods when conditions permit. The relationship between sleep disturbance and cognitive changes after stroke was further investigated. The pathological significance and molecular mechanisms of non-respiratory related sleep disorders secondary to stroke are not well-understood and need further study and confirmation.

## Data availability statement

The original contributions presented in the study are included in the article/supplementary material, further inquiries can be directed to the corresponding author/s.

## Author contributions

YZ: conceptualization, methodology, software, and writing—original draft preparation. XX and TZ: data curation and writing—original draft preparation. CZ: visualization and investigation. RL and SL: supervision. YY and CZ: software and validation. WY and XL: writing—reviewing and editing. All authors contributed to the article and approved the submitted version.

## Funding

This article was funded by Tianjin Key Medical Discipline (Specialty) Construction Project (No. TJYXZDXK-052B).

## Conflict of interest

The authors declare that the research was conducted in the absence of any commercial or financial relationships that could be construed as a potential conflict of interest.

## Publisher's note

All claims expressed in this article are solely those of the authors and do not necessarily represent those of their affiliated organizations, or those of the publisher, the editors and the reviewers. Any product that may be evaluated in this article, or claim that may be made by its manufacturer, is not guaranteed or endorsed by the publisher.
